# A Model for the Development of Hospital Beds Using Fuzzy Analytical Hierarchy Process (Fuzzy AHP)

**Published:** 2017-11

**Authors:** Ramin RAVANGARD, Mohammadkarim BAHADORI, Mehdi RAADABADI, Ehsan TEYMOURZADEH, Khalil ALIMOMOHAMMADZADEH, Fardin MEHRABIAN

**Affiliations:** 1. Health Human Resource Research Center, School of Management and Medical Information Sciences, Shiraz University of Medical Sciences, Shiraz, Iran; 2. Health Management Research Center, Baqiyatallah University of Medical Sciences, Tehran, Iran; 3. Research Center for Health Services Management, Institute for Futures Studies in Health, Kerman University of Medical Sciences, Kerman, Iran; 4. Dept. of Health Services Management, North Tehran Branch, Islamic Azad University, Tehran, Iran; 5. Dept. of Education and Health Promotion, School of Health, Guilan University of Medical Sciences, Guilan, Iran

**Keywords:** Health system, Military hospitals, Fuzzy analytical hierarchy process, Multi-criteria decision making

## Abstract

**Background:**

This study aimed to identify and prioritize factors affecting the development of military hospital beds and provide a model using fuzzy analytical hierarchy process (Fuzzy AHP).

**Methods:**

This applied study was conducted in 2016 in Iran using a mixed method. The sample included experts in the field of military health care system. The MAXQDA 10.0 and Expert Choice 10.0 software were used for analyzing the collected data.

**Results:**

Geographic situation, demographic status, economic status, health status, health care centers and organizations, financial and human resources, laws and regulations and by-laws, and the military nature of service recipients had effects on the development of military hospital beds. The military nature of service recipients (S=0.249) and economic status (S=0.040) received the highest and lowest priorities, respectively.

**Conclusion:**

Providing direct health care services to the military forces in order to maintain their dignity, and according to its effects in the crisis, as well as the necessity for maintaining the security of the armed forces, and the hospital beds per capita based on the existing laws, regulations and bylaws are of utmost importance.

## Introduction

The health systems around the world are faced with the weaknesses in the management and development, and their development should be paid special attention (1). In addition, poor management and lack of information for effective decision-making can lead to the lack of control, poor organization, and inefficiencies of hospitals. On the other hand, the most important function of all managers is timely decision making, and this can be achieved when the sufficient information about the considered issue is available and factors affecting the organizational development have been studied and determined (2). Hospitals are constantly confronted with the challenges related to the internal and external environmental forces such as demographic changes, the use of new and expensive technologies, changes in the health market and economic conditions, and health system reforms (3). These changes and improvements can cause their managers and administrators to be faced with challenges. This shows the necessity of reviewing, revising and paying attention to the hospitals (4).

In most countries, the health care costs have risen sharply in recent years. In spite of the limited population covered, hospitals spend the major part of the budget allocated to health sector (5). The formulation and implementation of the effective and appropriate strategies that are gradual and continuous and seek to make significant and fundamental developments and changes in the organization are called Organizational Development or Developmental Changes (6). Therefore, ignoring the factors influencing the development of hospital beds can result in wasting the organizational costs, prolonging the business processes, dissatisfying the stakeholders of how to serve and provide services and, finally, reducing the effectiveness and efficiency of the organization and its employees’ activities (7).

Due to lack of resources, the managers of health organizations around the world should decide on which resources should be invested in and which ones should not be focused on (8). In order to develop the hospital beds, managers and administrators should identify the barriers to development and attract the support of different unit managers to participating in the professional development in order to change the existing culture into lifelong learning (9). Factors such as policymaking, management, techniques of project implementation, development and dissemination, evaluation and assessment, as well as factors related to the society are important in the development of hospital beds (10).

One of the techniques used for decision making through the quantitative data is the analytic hierarchy process (AHP) (11). There are numerous studies on the use of AHP in the health sector, on the performance evaluation of medical records departments in the selected hospitals in Isfahan (12), on research priorities (13), and on analyzing the health inequalities (14),

Therefore, because one of the indicators of development in different countries is the efficacy of their health systems and one of the important decisions and measures were taken by the hospital managers is the development of hospital beds; this study aimed to identify and prioritize factors affecting the development of military hospital beds and provide a model using fuzzy analytical hierarchy process (Fuzzy AHP).

## Methods

This applied study was conducted in 2016 in Iran using a mixed method.

### First phase: The qualitative phase

In this phase, experts in the field of military health care system were involved in the study as the study population. The inclusion criteria were having at least five years’ job experience and having a Ph.D. degree. Overall, 22 experts participated in the present study. These studied persons were selected using the purposive sampling method. In this phase, three in-depth interviews with experts were conducted. Next, semi-structured interviews were conducted to collect data. The framework analysis and MAXQDA 10.0 software was used to analyze the collected data.

### Second phase: The fuzzy analytical hierarchy process (Fuzzy AHP)

A questionnaire was used in order to determine the degree of importance of factors affecting the ranking. In other words, the weights of factors were determined through designing a questionnaire and performing pairwise comparisons through using Fuzzy AHP. The factors and subfactors were scored and rated using qualitative scales and their related triangular fuzzy numbers (Table 1). Written informed consent was obtained from all participants in this study and all of them were assured of the confidentiality of their responses. This research project was approved by the Ethical Committee of Baqiyatallah University of Medical Sciences.

**Table 1:** The qualitative scales and their related triangular fuzzy numbers (38)

***Definitions***	***Preferred row to column***	***Preferred column to row***
Equally important	(1, 1, 1)	(1, 1, 1)
Equally important to moderately more important	(1, 2, 3)	(0.33, 0.5, 1)
Moderately more important	(1, 3, 5)	(0.2, 0.33, 1)
Moderately more important to strongly more important	(3, 4, 5)	(0.2, 0.25, 0.33)
Strongly more important	(3, 5, 7)	(0.14, 0.2, 0.33)
Strongly to very Strongly more important	(5, 6, 7)	(0.14, 0.17, 0.2)
Very Strongly more important	(5, 7, 9)	(0.11, 0.14, 0.2)
Very Strongly to extremely more important	(7, 8, 9)	(0.11, 0.13, 0.14)
Extremely more important	(7, 9, 9)	(0.11, 0.11, 0.13)

To determine the rankings and degree of importance of factors affecting the development of hospital beds, the fuzzy analytical hierarchy process (Fuzzy AHP) was used and the Expert Choice 10.0 software was applied.

### Fuzzy AHP

The concepts and definitions of Fuzzy AHP are described based on the Extent Analysis (EA) method. Consider two triangular numbers *M̃*_ 1_= (l_1_,m_1_,u_1_) and *M̃*_ 2_=(l_2_,m_2_,u_2_).

Their arithmetic operators are defined as follows:

$$\begin{array}{c} M_{1}+M_{2}=\left(l_{1}+l_{2},m_{1}+m_{2},u_{1}+u_{2}\right)\\ M_{1}*M_{2}=\left(l_{1}*l_{2},m_{1}*m_{2},u_{2}*u_{2}\right)\\ M_{1}^{-1}=\left(\frac{1}{u_{1}},\frac{1}{m_{1}},\frac{1}{l_{1}}\right),M_{2}^{-1}=\left(\frac{1}{u_{2}},\frac{1}{m_{2}},\frac{1}{l_{2}}\right) \end{array}$$

In the EA method, for each row of the pair-wise comparisons matrix, the value of *S_ K_*, which is a triangular number, is calculated as follows:

$$S_{k}=\sum_{j=1}^{n}M_{kl}*{\left[\sum_{i=1}^{m}\sum_{j=1}^{m}M_{ij}\right]}^{-1}$$

Where k is the number of rows, and i and j are the options and indices, respectively.

In this method, after calculating the S_k_’s, the level of their magnitude relative to each other should be determined. In general, if M_1_ and M_2_ are two triangular fuzzy numbers, the magnitude level of M_1_ relative to M_2_, which is denoted by V (M_1_≥M_2_), is defined as follows:

$$\begin{array}{l} \{\begin{array}{lll} V(M_{1}\geq M_{2})=1 & m_{1}\geq m_{2} & \\ \text{V}(M_{1}\geq M_{2})=\mathrm{hgt}(M_{1}\cap M_{2}) & & \\ & & \text{Otherwise} \end{array}\\ \mathrm{hgt}(M_{1}\cap M_{2})=\left(\bar{u_{1}}-l_{2}\right)+(m_{2}-m_{1}) \end{array}$$

The magnitude level of a triangular fuzzy number relative to k other triangular fuzzy numbers are calculated by the following equation:

$$V\left(M_{1}\geq M_{2},\ldots ,M_{k}\right)=Min[V\left(M_{1}\geq M_{2}\right),\ldots ,V\left(M_{1}\geq M_{k}\right)]$$

In the EA method, he weights of indices in the pairwise comparisons matrix are calculated by:

$$W^{ó}\left(X_{i}\right)=Min\{V(S_{i}\geq S_{k})\},\;\;\;\;k=1,2,\ldots ,n,k\neq i$$

Therefore, the vector of indices’ weight, which is the vector of the fuzzy AHP non-normal coefficients, is as follows:

$$W^{ó}={\left[W^{ó}\left(c_{1}\right),W^{ó}\left(c_{2}\right),\ldots ,W^{ó}\left(c_{n}\right)\right]}^{T}$$

## Results

Most of the experts participating in the present study had 5–10 yr management experience and studied in the field of Health Services Management. Their mean age was 44±1.7.

The main factors influencing the development of military hospital beds included geographic situation, demographic status, economic status, health status, health care centers and organizations, financial and human resources, laws and regulations and bylaws, and the military nature of service recipients. The military nature of service recipients (S=0.249) and economic status (S=0.040) received the highest and lowest priorities, respectively (Table 2). The highest and lowest priorities of subfactors have shown in Table 3.

**Table 2:** Prioritization of the factors affecting the development of military hospital beds from the studied experts’ viewpoints using Fuzzy AHP

***Codes***	***Factors***	***Scores (S)***	***Priorities***
1	Geographic situation	0.109	4
2	Demographic status	0.087	6
3	Economic status	0.040	8
4	Health status	0.080	7
5	Health care centers and organizations	0.154	3
6	Financial and human resources	0.096	5
7	Laws and regulations and bylaws	0.184	2
8	The military nature of service recipients	0.249	1

**Table 3:** The priorities of subfactors affecting the development of military hospital beds

***Factors***	***Subfactors***	***Scores (S)***	***Priorities***
**F1: Geographic situation**	C1: Province distance from the major and big cities	0.055	5
	C2: Position and situation of the health care center and organization	0.260	2
	C3: Boundary conditions of the province	0.123	4
	C4: Distance from the nearest equipped military health care center and organization	0.200	3
	C5: Population density in the urban and rural areas	0.361	1
**F2: Demographic status**	C6: Need for full implementation of the Adaptation Plan	0.160	3
	C7: Being a host province	0.119	4
	C8: Sex and age distribution of the urban and rural population	0.088	5
	C9: Annual population growth rate	0.088	5
	C10: Rates of births and deaths	0.054	6
	C11: Rates of illiteracy and literacy	0.035	7
	C12: Cultural values, social and political characteristics	0.201	2
	C13: Population of the considered province or city/pato-geographic status	0.255	1
**F3: Economic status**	C14: High cost of civilian health care services	0.075	6
	C15: Economic evaluation of providing services	0.319	1
	C16: Sources of income	0.176	2
	C17: Per capita income per year	0.102	4
	C18: Employment rate	0.045	8
	C19: Rate of inflation	0.060	7
	C20: Interest rate for investment in the health insurance	0.137	3
	C21: Being an industrial province	0.085	5
**F4: Health status**	C22: Common diseases in the military forces	0.136	3
	C23: Understanding the epidemiology of diseases and the number of patients in the society	0.049	7
	C24: Vulnerable groups	0.099	5
	C25: Vulnerability to common diseases in the region	0.075	6
	C26: Life expectancy	0.134	4
	C27: Health Promotion	0.186	2
	C28: Equity in health care	0.322	1
**F5: Health care centers and organizations**	C29: Existence of empty capacities in the province	0.050	7
	C30: Training and research services	0.131	4
	C32: Lack of appropriate health care centers and organizations and the ease of service delivery to the armed forces	0.325	1
	C33: Bed occupancy rate	0.165	2
	C34: Admission rate per bed in each year	0.102	5
	C35: Average length of stay	0.092	6
	C36: Existence of physical spaces	0.135	3
**F6: Financial and human resources**	C37: Status of the existing health technologies	0.047	6
	C38: Resources available to build the new hospital wards	0.197	3
	C39: Having the potential for developing the existing hospital beds	0.242	2
	C40: Existence of modern and updated equipment	0.071	5
	C41: Having extensive specialty and subspecialty facilities and equipment	0.091	4
	C42: Having access to medical and paramedical personnel	0.353	1
**F7: Laws and regulations and bylaws**	C43: Health policies at the national level	0.115	3
	C44: Empathy among the commanders of military units stationed in the province	0.444	1
	C45: Having appropriate intersectoral cooperation in the province	0.069	5
	C46: Cooperation among province authorities in setting up a specialty hospital	0.048	6
	C47: Hospital beds per capita approved by the General Staff of the Armed Forces	0.085	4
	C48: Hospital beds per capita approved by the Ministry of Health	0.198	2
**F8: The military nature of service recipients**	C49: Maintaining dignity and increasing job satisfaction	0.087	4
	C50: Maintaining the dignity of military personnel	0.081	5
	C51: Necessity for paying attention to the military families’ well-being	0.057	6
	C52: Necessity for direct treatment of the military forces, especially in the crises	0.258	2
	C53: Necessity for maintaining the security of the armed forces	0.293	1
	C54: Necessity for conducting special clinical studies on the military forces	0.041	7
	C55: Number of military forces in the province	0.183	3

## Discussion

An overview of the status of Iran hospitals indicates that most of them are faced with increased demand, overcrowding and patients’ dissatisfaction with access to services and their quality. On the other hand, the construction, set-up, and management of hospitals are very expensive, and building a hospital requires huge initial investments (15).

According to the results, among the factors influencing the development of military hospital beds, the military nature of service recipients received the highest priority (S=0.249). Paying attention to the nature of the service recipients is important because obtaining information through the study of service recipients is a successful method for strategic assessments and developments of health services (16). Concerning the military nature of service recipients, the necessity for maintaining the security of the armed forces (S=0.293) received the highest priority. Factors such as respecting the patients’ privacy, safety, and personality were the most important subfactors from the managers and experts’ viewpoints (17). In addition, in the military nature of service recipients, factors such as maintaining dignity and increasing job satisfaction and maintaining the dignity of military personnel were the other important subfactors. In addition, in the military nature of service recipients, factors such as maintaining dignity and increasing job satisfaction and maintaining the dignity of military personnel were the other important subfactors. The best and most important indicator to measure service quality is the level of service recipients’ satisfaction (18).

Regarding the geographic situation, the subfactors of population density in the urban and rural areas (S=0.361) and position and situation of the health care center and organization (S=0.260) had the highest priorities. Factors such as the distance from the patients’ place of residence (19), hospital location and how to access the hospital (20, 21), and traveling to receive care and services (22, 23) are important to affecting the development of hospital beds. The geographic situation of health care organizations can make possible the hospital beds development through providing easy access for patients.

About the demographic status factor, the subfactors of the population of the considered province or city/pato-geographic status and cultural values, social and political characteristics had the first and second priorities among eight subfactors. Physical, socioeconomic, cultural and political factors had effects on the development of healthcare and hospital beds (24) which is in line with the results of the present study. Furthermore, factors such as the age and sex distribution and social status had effects on the development of health centers and hospital beds (25–28).

Relating to the economic status, the highest priorities were related to the economic evaluation of providing services (S=0.319) and sources of income (S=0.176). Households’ income had effects on the economic status (23). Furthermore, the high cost of civilian health care services (S=0.075) obtained the lowest priority, although the price of services is a major factor influencing the development of hospital beds (23, 27). The differences between the results of the present study and others can be due to the low cost of services provided for the individuals covered by the armed forces insurance (29).

Pertaining to the health status, the equity in health care had the highest priority (S=0.322). Today, health equity and eliminating the inequity in the health sector are one of the main concerns of the health systems in the world, especially in the developing countries (30). In this regard, the WHO has emphasized the need for measuring equity in the distribution of resources because access to health care is a fundamental right of all human beings, disparities in the geographical distribution of health resources can cause some problems for access to health services (31).

The subfactor of vulnerability to common diseases in the region had the lowest priority among the subfactors of health status. The regions vulnerable to certain diseases had obtained the fourth priority (32). However, paying special attention to the vulnerable groups in the society when evaluating the social needs, and developing compensatory mechanisms to solve the health problems of people who are in poor health are very important (33).

Referring to the healthcare centers and organizations, the subfactors of bed occupancy rate, existence of physical spaces, training and research services, admission rate per bed in each year, average length of stay, and existence of empty capacities in the province are important. Factors such as the number of beds and services (19) and the admission rate per bed (34) were important factors in developing hospital beds.

Concerning the financial and human resources, having access to medical and paramedical personnel (0.353) had obtained the highest priority. Having the good and skilled physicians and staff had effects on the development of hospital beds (35). Although in the present study, the existence of modern and updated equipment had received a low priority, factors such as the existence of advanced equipment and specialized facilities are important factors for the development of hospital beds (19, 27). Relating to the laws, regulations, and by-laws, the empathy among the commanders of military units stationed in the province had the highest priority. Paying attention to the empathy in the hospitals was very important (36).

The fuzzy AHP, compared to AHP and the statistical methods of prioritization, has higher precision and certainty. Although in AHP the experts compare the options using their competencies and intellectual assets, it may not fully reflect the style of human thinking. However, the use of fuzzy numbers is more compatible with human linguistic expressions. Therefore, the decisions can be made better and more accurately in the real world using fuzzy numbers (37).

## Conclusion

The importance of “The military nature of service recipients” and “Laws and regulations and bylaws” in the development of hospital beds, these two factors should be paid special attention in the policy- and decision-making. Providing direct health care services to the military forces in order to maintain their dignity, and according to its effects in the crisis, as well as the necessity for maintaining the security of the armed forces, and the hospital beds per capita based on the existing laws, regulations and bylaws are of utmost importance.

## Ethical considerations

After the researchers explained the purpose and procedures of the study to the studied managers participating in the present study, they consented to participate in the study. In addition, the ethical issues (including plagiarism, misconduct, data fabrication and/or falsification, double publication and/or submission, redundancy, etc.) have been completely observed by the authors.
